# Reconstruction of Medial Wall Blowout Fracture Defect with a Combination of Resorbable Meshed Plate and Cancellous Bone Allograft

**DOI:** 10.1155/2019/2656503

**Published:** 2019-10-15

**Authors:** Jongweon Shin, Song I Park, Yunsup Hwang, Ho Kwon, Hyung-Sup Shim

**Affiliations:** ^1^Department of Plastic and Reconstructive Surgery, Yeouido St. Mary's Hospital, College of Medicine, The Catholic University of Korea, Seoul, Republic of Korea; ^2^Department of Plastic and Reconstructive Surgery, Uijeongbu St. Mary's Hospital, College of Medicine, The Catholic University of Korea, Uijeongbu-si, Gyeonggi-do, Republic of Korea; ^3^Department of Radiology, Uijeongbu St. Mary's Hospital, College of Medicine, The Catholic University of Korea, Uijeongbu-si, Gyeonggi-do, Republic of Korea; ^4^Department of Plastic and Reconstructive Surgery, St. Vincent's Hospital, College of Medicine, The Catholic University of Korea, Suwon-si, Gyeonggi-do, Republic of Korea

## Abstract

**Background:**

Various materials are available for the reconstruction of bone defects in cases of medial wall blowout fracture. This study was conducted to assess the efficacy of the combination of a resorbable meshed plate and cancellous bone allograft.

**Methods:**

From March 2014 to March 2017, a total of 111 patients were evaluated. Sixty-three patients received reconstruction surgery with porous polyethylene plates (control group) and the other forty-eight patients underwent operation with a resorbable meshed plate plus allogenic cancellous bone (combined group). The results were assessed by exophthalmometric measurements, width, and volume discrepancies as compared with the unaffected orbit, and operation time.

**Results:**

The difference in exophthalmometric measurements between the affected and unaffected orbits were 0.94 ± 0.70 mm in the control group and 1.05 ± 0.73 mm in the combined group without statistical significance (*p* = 0.425). In the analysis of computed tomography images, the width discrepancy was 1.55 ± 0.86 mm and 1.08 ± 0.69 mm, respectively (*p* = 0.003); however, the volume discrepancy demonstrated no statistically significant difference (2.58 ± 1.40 cm^3^ versus 2.20 ± 1.80 cm^3^; *p* = 0.209). Operation time was significantly shorter in the combined group as compared with the control group (43.0 ± 7.0 versus 38.3 ± 7.0 minutes; *p* = 0.001).

**Conclusion:**

The combination material composed of resorbable meshed plate and cancellous bone allograft made reconstruction surgery of medial wall blowout fracture easier and quicker to perform with long-lasting results.

## 1. Introduction

Blowout fracture is defined as a fracture that involves the orbital walls, especially the medial wall and/or orbital floor [1]. It may be one of the most commonly encountered facial bone fractures by physicians because of the exposed position of the globe and the relatively thin properties of the orbital bone [2]. There have been numerous studies suggesting that the medial wall is the most commonly affected area among other types [3].

The management of medial wall blowout fracture remains controversial. Some surgeons suggest that small-sized defects are not a definite indication for operation. However, functional and aesthetic sequelae, such as diplopia or enophthalmos, could also occur as they do in orbital floor blowout fractures [4]. Therefore, if the patient presents with diplopia or enophthalmos and/or extensive fracture is observed in computed tomography (CT) images, then surgical reconstruction is recommended [2].

Various substances such as autogenous materials (e.g., autologous bone, cartilage, and fascia), allogenic materials, nonresorbable alloplastic materials (e.g., titanium, porous polyethylene, hydroxyapatite), and resorbable alloplastic materials (e.g., poly-L-lactic acid, polyglycolic acid, polyglactin, composite polymers) can be used in the reconstruction of medial orbital wall fractures and defects [1, 5, 6]. Recently, resorbable alloplastic materials have become one of the most popular implant options because of their safety, simplicity, and effectiveness without donor site morbidities or permanent residues. However, debate remains about their long-term efficacy of enophthalmos prevention due to their resorbing nature.

Therefore, we attempted to evaluate a new surgical technique by using a resorbable meshed plate with allogenic cancellous bone. The flexible resorbable meshed plate was applied to enable easy insertion via a relatively small visual field of transcaruncular incision, while allogenic cancellous bone was chosen as a bone substitute because of not only its abilities for osteoconduction and osteoinduction but also for its elasticity and volume-filling effect that mimics the ethmoid sinus space.

For this study, we compared this new combination material with porous polyethylene sheeting, which is one of the most widely used permanent materials for the reconstruction of medial wall blowout fracture defects. We herein present our experience along with long-term follow-up results.

## 2. Patients and Methods

### 2.1. Ethical Statement

This study was approved by the institutional review board of the catholic university of Korea (no. UC16DISI0065). All data were analyzed anonymously and according to the principles set forth in the declaration of Helsinki (1975, revised in 2008).

### 2.2. Patients

From March 2014 to March 2017, a total of 273 patients with medial wall blowout fracture were enrolled. A thorough review of patients' past trauma and medical history, ophthalmic examinations including visual acuity, intraocular pressure, extraocular muscle function and other associated ocular complications was done. Fourty six patients were excluded according to the following criteria; age younger than 18 years, previous trauma history involving orbital bones, combined facial bone fractures including orbital floor, severe underlying diseases that could delay bone healing, bilateral fractures, and emergent ocular complications such as significant change in visual acuity, increased intraocular pressure, globe rupture and retrobulbar hemorrhage. As we performed the surgical intervention only when patients presented diplopia with evidence of extraocular muscle entrapment on CT scan, enophthalmos more than 2 mm, or fracture area size of more than 1 cm^2^ in CT images, additional 74 patients were also excluded. Among the others, 89 patients received reconstruction surgery with porous polyethylene plates (control group) and the other 64 patients received operation with resorbable meshed plate plus allogenic cancellous bone (combined group). Because patients who underwent CT scan at least 12 months after their operation were evaluated, only 63 and 48 patients were ultimately included in this study, respectively.

### 2.3. Description of Reconstruction Materials

The following are two main elements of our new combination method.

First, the resorbable meshed plate implant used was Osteomesh™ (Osteopore International, Singapore), which is a bioresorbable implant often used in craniofacial surgery to fill surgical defects. It is made of polycaprolactone, which will degrade and resorb fully in vivo by hydrolysis and then is metabolized by the body over a period of 18 to 24 months. The mesh offers a rigid yet flexible scaffold with enough mechanical strength that supports bone in-growth. It degrades as bone regeneration and is replaced by autologous bone [7].

Second, the alloplastic cancellous bone used was Genesis Sponge™ (Hans Biomed Co., Seoul, Republic of Korea), a sponge-type allograft transplant material that employs the demineralized cancellous bone to induce proliferation of mesenchymal cells and osteoblast differentiation to help form normal bone. It is rapidly integrated into the recipient site and has high osteoconduction and osteoinduction properties. There are two types: block and chip. We used the former one to establish a three-dimensional (3D) structure of the defect easily [8].

**Figure 1:**
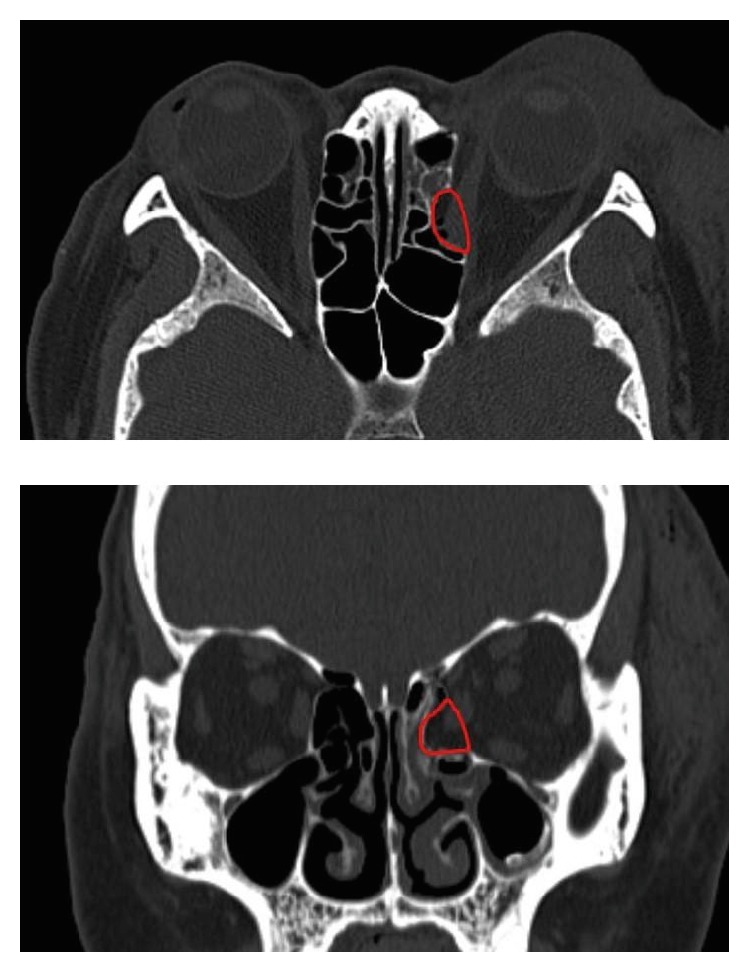
3D shape of the defect was estimated based on CT images. Red line indicates the defect area to be replaced by cancellous bone allograft with inlay implantation method.

**Figure 2:**
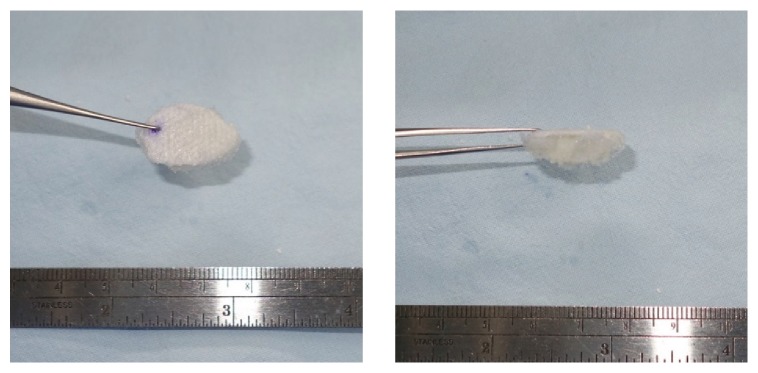
The reconstruction material used in the combined group was composed of resorbable meshed plate and allograft cancellous bone. Rehydrated bone sponge was sculpted according to the 3D shape of the defect based on CT images and was attached to the resorbable plate with fibrin glue.

In the operation room, the resorbable meshed plate was trimmed with scissors to exactly fit into the medial orbital wall defect. Then, the cancellous bone blocks were stacked upon the resorbable plate using fibrin glue after eight minutes of rehydration. The shape was sculpted according to the 3D structure of the fracture to fill the bony defect (Figure 1). The overall shape could be achieved by trimming with scissors without difficulty as the cancellous bone chips, once rehydrated, become soft as a sponge-like material. The resorbable plate and cancellous bone complex could be easily flexed and smoothly inserted through the small transcaruncular incision due to its high flexibility and elasticity (Figure 2).

### 2.4. Surgical Procedure

Under general endotracheal anesthesia, a transcaruncular incision was made to approach the medial wall of the orbit. Deep to the posterior lacrimal crest, a periosteal incision was performed and subperiosteal dissection was continued to expose the fracture site. After nonvitalized bone segments were debrided, the anterior, cranial, caudal, and posterior margins of bony defect were identified.

For establishing an onlay graft using porous polyethylene sheeting, the dissection was continued slightly wider than the fracture margin, because it needs to be placed on the unfractured bone and secured. If the fracture was too large to expose all margins, at least three margins should be identified. Then, the polyethylene plate was trimmed and inserted.

In the combined group, the combination material described in the previous section was used. Because the cancellous bone was inserted into the ethmoidal sinus and the resorbable meshed plate was placed just fit to the defect, dissection over the fracture margins was not necessary. After visualization of the fracture area, the fabricated combination material was flexed using forceps and inserted into the defect. After the insertion, due to its elasticity, the combination material was automatically unfolded to fill in the orbital wall defect.

Forced duction test was performed to confirm no extraocular muscle or soft tissue entrapment after the implant insertion, and the mucosal closure was done with an absorbable suture.

### 2.5. Evaluation of Reconstruction Results

Pre- and postoperative clinical symptoms such as binocular visual acuity, discomfort during eyeball movement, extraocular muscle movement limitations, and diplopia were documented. Exophthalmometric measurements of both eyes were measured by hertel exophthalmometry and recorded.

**Figure 3:**
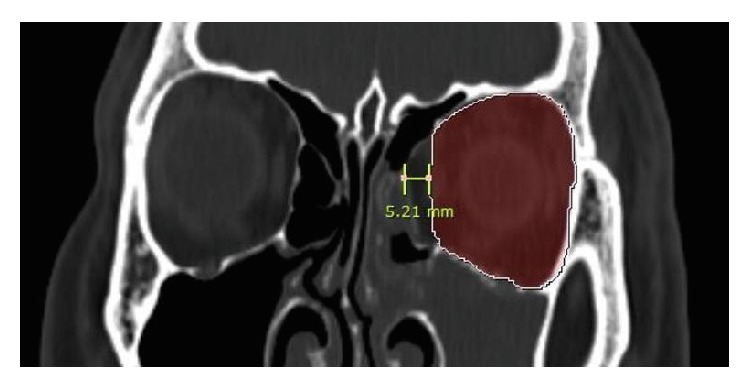
To evaluate width discrepancy, the mirror image of the patient's noninjured orbit was used as a criterion. The imaginary medial wall based on the mirror image of the patient's noninjured orbit was drawn and the highest width discrepancy was measured.

**Figure 4:**
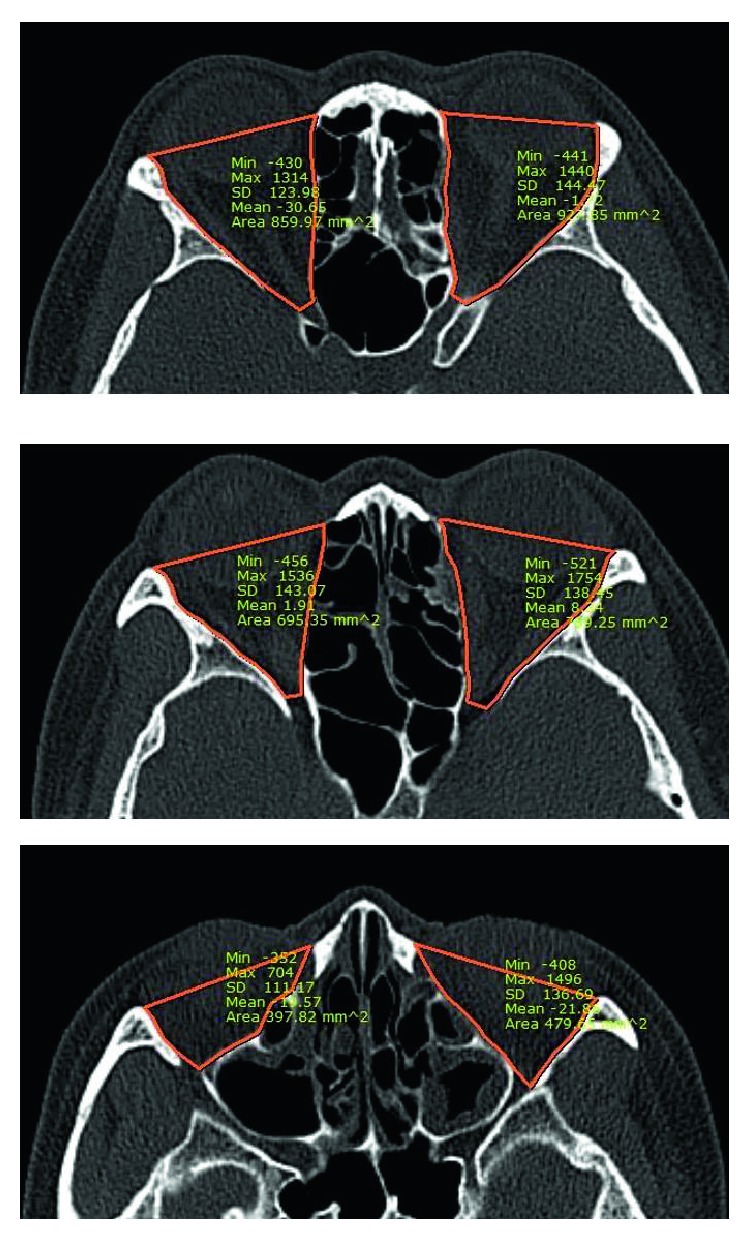
To calculate orbital volume discrepancy, the areas of both orbits were measured by image processing program and all areas were added and compared.

All patients underwent 3D CT imaging obtained at a thickness of 1 mm for the evaluation of accurate fracture margin shape and bony defect. Postoperative CT scans were performed at 1, 3, 6, and 12 months from the surgery and images taken at least 12 months after the operation were evaluated for results. The mirror image of the patient's uninjured orbit was used as a criterion to estimate the postoperative width discrepancy. The imaginary medial wall was drawn in the coronal section of the affected orbit and the largest width difference was measured. Every coronal section of each patient was evaluated, and the highest value was selected (Figure 3). To assess the volume discrepancy, a picture-archiving communication system (PACS; Marosis m-view; Infinitt Healthcare, Seoul, Republic of Korea) with an automated region of interest was used. The calculation was done as previously described in other studies [9–12]. The area was measured by a freehand drawing cursor tracing the orbital wall in the axial section. The anterior margin of the orbit was defined as the line drawn from the anterior lacrimal crest of the maxilla to the lateral orbital rim. Volume was calculated by adding all the areas and multiplying them by the section thickness (1 mm in this study) (Figure 4). All width and volume discrepancy measurements were done twice by a single head and neck radiologist (Y. H.) and then the results were averaged to reduce measurement errors. Operation time from mucosal incision to skin closure was also recorded in all cases.

### 2.6. Statistical Analysis

The Statistical Package for the social sciences version 24.0 for windows software program (IBM Corp., Armonk, NY, USA) was used for statistical analysis. Mean data were analyzed with the independent samples *t*-test and categorical variables were evaluated by pearson's chi-square test. Statistical significance was set at *p* ≤ 0.05.

## 3. Results

A total of 111 patients were enrolled in this study. 63 patients were assigned to the control group and the other 48 patients were placed in the combined group. The male to female ratios for each group was 2 : 1 versus 1.52 : 1 and the mean ages were 41.2 (range: 18–68) and 38.5 (range: 18–70) years, respectively. The mean follow-up periods were 19.3 months (range: 12–30) in the control group and 15.5 months (range: 12–22) in the combined group. Etiologies of the fractures included assault (43%), traffic accident (19%), fall (16%), sports injury (13%), and industrial injury (10%) in the control group, with values of 48%, 21%, 15%, 10%, and 6% for the same in the combined group, respectively. There were no statistically significant differences in male to female ratio, mean age and etiology between the two groups (Table 1).

All patients received a meticulous ophthalmic functional examination including measurement of visual acuity, intraocular pressure, extraocular muscle function and diplopia test, and exophthalmometry. Mean visual acuity in both groups were 0.87 ± 0.39 and 0.94 ± 0.45 by Landolt ring test (*p* = 0.375) and mean intraocular pressure were 14.24 ± 3.62 mmHg and 13.40 ± 3.67 mmHg, respectively (*p* = 0.230). A total of 16 patients presented extraocular movement dysfunction or diplopia in the control group (16/63, 25.40%) and 17 in the combined group (17/48, 35.42%), and this difference was not statistically significant (*p* = 0.253). Preoperative exophthalmometry discrepancy between the injured eye and the noninjured eye were 1.11 ± 0.69 mm and 1.19 ± 0.67 mm, respectively (*p* = 0.505). Based on preoperative CT images, the fracture area size was calculated in both groups. It was measured as 1.75 ± 0.52 cm^2^ in the control group and 1.85 ± 0.66 cm^2^ in the combined group and showed no significant difference (*p* = 0.347). These preoperative evaluations are summarized in Table 2.

Postoperative results were evaluated by exophthalmometry, CT images and the length of the operation time. Exophthalmometric measurements were obtained and compared at least 12 months after surgery. The discrepancy between the injured eye and the unaffected eye was 0.94 ± 0.70 mm in the control group and 1.05 ± 0.73 mm in the combined group. This showed no significant difference (*p* = 0.425). In the analysis of postoperative CT images, the highest width discrepancy between the reconstructed medial wall and the mirror image of the uninjured eye was evaluated. The findings were 1.55 ± 0.86 mm in the control group and 1.08 ± 0.69 mm in the combined group, and this showed a significant difference (*p* = 0.003). However, the volume discrepancy between the two groups demonstrated no statistically significant difference (2.58 ± 1.40 cm^3^ versus 2.20 ± 1.80 cm^3^; *p* = 0.209). The length of the operation time was also recorded. As compared with the control group, operation time was significantly shorter (43.0 ± 7.0 versus 38.3 ± 7.0 minutes;*p* = 0.001) in the combined group. All postoperative results are demonstrated in Table 3.

No patients suffered from specific complications. Nine patients in the control group and seven in the combined group complained temporary diplopia after surgery but all symptoms resolved within 1 month. The other patients were discharged without complications and there were no permanent complications such as delayed diplopia, enophthalmos, and wound infection.

### 3.1. Case 1

**Figure 5:**
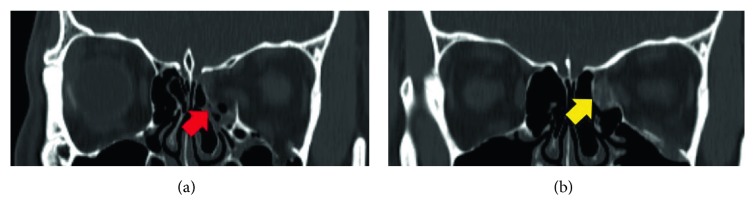
CT images of the case 1 patient. Preoperative CT scan shows fracture and bone defect in the medial wall of the left orbit (a, red arrow). The orbital contents are well maintained inside the orbit by combination plate. Note the weak signal intensity of bone formation in the postoperative CT scan obtained at 12 months after operation (b, yellow arrow).

A 27-year-old male patient visited the emergency department with left periorbital swelling due to a traffic accident. He complained of mild discomfort when he gazed bilaterally but no diplopia. In CT scan images, a 1.45 cm^2^-sized fracture and bone defect were observed in the medial wall of the left orbit (Figure 5). At six days after injury, he underwent operation with resorbable meshed plate and allogenic cancellous bone. After 12 months of follow-up, exophthalmometric measurement discrepancy between the affected and unaffected eyes was reduced from 2 to 0.5 mm. The CT images showed weak signal intensity of bone formation. The highest width discrepancy was 1.1 mm, and volume discrepancy was calculated as 1.8 cm^3^. He presented no enophthalmos or other complications during the follow-up period.

### 3.2. Case 2

**Figure 6:**
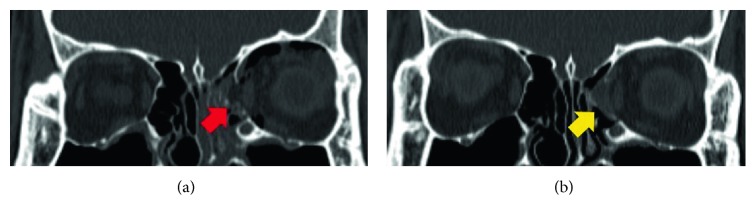
CT images of the case 2 patient. Initial fracture and defect (a, red arrow) was well-reduced by a combination plate of resorbable meshed plate and allogenic bone substitute (b, yellow arrow).

A 29-year-old male presented with diplopia upon his arrival at the emergency department. The fracture had occurred by fist injury and he was operated on two days after the assault with combined implant. Initial fracture area size was measured as 1.81 cm^2^. The 15-month follow-up CT showed a well-reduced state with the highest width discrepancy being 1.06 mm and volume discrepancy being 0.8 cm^3^(Figure 6). He complained of no problems during the follow-up period except temporary diplopia, which resolved spontaneously within two weeks after surgery.

## 4. Discussion

Blowout fracture is one of the most frequently encountered facial bone fractures, although true incidence is unknown [2, 13]. Some studies have shown that the medial wall is the most commonly affected area in comparison with other parts of the orbit [3]. Rapid development in medical imaging technology over recent years, numbers of diagnosis and operations of medial wall blowout fractures have increased.

Various materials are available for the reconstruction of medial wall blowout fracture defects. Theoretically, using autologous bone is ideal, but it has many drawbacks such as donor site morbidity, unpredictability due to various degrees of resorption, limited availability, and patient refusal. These limitations have urged the development of alternative alloplastic materials. Nonresorbable plates such as porous polyethylene and titanium can be used as a second choice. However, they have limitations of a potential risk of infection and late presentation of extrusion. Impingement of soft tissues and the need for a relatively large incision were also disadvantages [4].

Resorbable materials are additionally available for filling the defect. They have the ability of supporting the orbital soft tissue from herniation and degradation by hydrolysis while leaving connective tissue in time. Their safety, simplicity, and effectiveness without donor site morbidity or permanent residue make them popular. However, a debate remains about their long-term efficacy because of their low strength and limited durability [1, 4].

To enhance the advantages of each material and compensate for their disadvantages, we decided to combine two materials. One is a flexible bioresorbable meshed plate and the other is alloplastic cancellous bone. We already reported our patients' data regarding orbital floor blowout fractures receiving operation with the combined materials [1]. We reconstructed a large orbital floor defect with the combination of a resorbable plate and artificial bone substitute in that study and obtained desirable results. However, when we applied this combination to medial wall blowout fracture defects, we confronted some problems. First, the resorbable plate was too rigid to pass through a transcaruncular incision. A transcaruncular incision is relatively small and there was a limit to the size of object able to be inserted. Second, for the same reason, dissection was limited for covering of the defect with this onlay-type implantation.

Therefore, we decided to change the resorbable plate from a rigid one to a flexible meshed plate. This could be flexed well with forceps grasping and, after passing the incision, it unfolded due to its elasticity. In addition, we applied alloplastic cancellous bone (Genesis Sponge™)for the inlay graft. After rehydration, it changed from a hard block into a sponge-like material. Therefore, it is simple to establish a 3D structure of the bone defect according to the preoperative CT images and easy to fold it to pass the small incision. As a result, this new combination enabled operators to insert the material easily due to folding capacity and, after insertion, it regained its original form because of elasticity. The cancellous bone unfolded according to the 3D structure of the bone defect and supported the resorbable plate to maintain its position—in other words, the plate acted as a cap. Therefore, surgeons were able to perform reduction surgery with less dissection using this inlay-type implantation method as compared with onlay grafting through a relatively limited visual field. This contributed to safer surgery without injury to the normal surrounding tissues and a reduction in operation time.

Furthermore, its long-term results were also desirable. As compared with the traditional method using polyethylene plates, there were no statistically significant differences in exophthalmometric measurement and postoperative volume discrepancy without notable complications. However, width discrepancy evaluation showed superior results versus polyethylene plate. This may be due to the elasticity of Genesis Sponge™. The nature of the resorbing process of grafted allogenic cancellous bone is not well-identified, but it maintained its initial position for at least three months in CT images according to our observations. Although not all bone substitutes formed new bone, the bony structure remained to support the resorbable plate to be successfully substituted by fibrous connective tissue in its original position, which we can identify in postoperative CT images (Figures 4 and 5). The medial wall of the orbit is a structure supported by many ethmoid air cells and multiple septa between them [14]. If the defect of the medial wall is covered by the onlay plates with septa left destroyed, the durability can be significantly decreased due to its structural instability. Hence, we used cancellous bone allograft (Genesis Sponge™) to act as the supporting material, like septa by reproducing its honeycomb structures, to push the orbital contents out from the ethmoid sinus, and maintain its structure for at least three months. This may make difference versus polyethylene plates. Nevertheless, width discrepancy did not lead to exophthalmometric measurement and postoperative volume differences in our study. While the volume values are different in various articles, an around 1.25 cm^3^ increase in the orbital volume results in an increase in enophthalmos of 1 mm. This outcome may be because the amount of displacement of the orbital medial wall was too little to make a difference between the two groups in exophthalmometric measurement or orbital volume discrepancy [9, 15–18].

Various composite implants have been introduced to reconstruct orbital wall defects. The typical most commonly used material is titanium-reinforced porous polyethylene (e.g., Synpor®) [19, 20]. Titanium mesh offers high strength and stability, while polyethylene allows vascular component ingrowth into the pores with a smooth surface. However, its rigidity is a major limitation in the reconstruction of medial wall blowout fracture. Resorbable implants mixed with biological ceramics (e.g., hydroxyapatite) are also used [21, 22]. For example, Osteotrans™ is a resorbable implant composed of poly L-lactic acid and hydroxyapatite. It is provided in various shapes and it is widely used in the craniofacial reconstruction field. Although it can be a good option for the reconstruction of orbital floor defects, it is stiffer than other pure resorbable implants for the use in medial wall blowout fracture reconstruction. Newly synthesized products including bioactive glass [23], nylon foil [24], bone morphogenetic protein-loaded hydrogel [25], and bone marrow-coated polycaprolactone [26] have been introduced recently. However, most of them are still in experimental stages, need large scale studies and further rules and regulations in terms of safety. By combining the resorbable meshed plate and the alloplastic cancellous bone, our combination method has the advantages of each material with some degree of rigidity, flexibility, and elasticity. In addition to that, it can be easily trimmed into a desired shape and the safety of these materials has been already proven in various fields.

As mentioned above, the additional advantage of using our combination is shortened operation time. This may be due to no need for extensive dissection through a limited incision area. In the preliminary study, the surgery took more time with the combined plate because additional time was needed to fabricate. The fabrication process included hydration of the alloplastic cancellous bone, molding according to the 3D structure of the defect, and fixating the bone to the meshed plate. However, an assistant made the combined plates based on preoperative CT images, while the dissection was done by the operator simultaneously and, as the assistant practiced more and more, the time for fabrication was shortened and the final operation time in the combined group was shorter than that in the control group. With a more trained assistant or 3D printing technology in the future, further shortening of the operation time will be able to be achieved.

Despite the excellent properties of resorbable plates and bone allografts, the materials are expensive. Therefore, the cost-effectiveness of our combination can be a burden to patients involved. Another major drawback of our technique is that it is limited to only medial wall blowout fracture reconstruction. Our combination materials are designed to fill into the bony defect formed by ethmoid air cells. Therefore it is not applicable in cases of blowout fractures involving the inferomedial wall or the floor. In such cases, other materials should be used instead.

In conclusion, reconstruction of orbital medial wall fracture with resorbable meshed plate and cancellous bone allograft can provide long-lasting results without significant complications. Furthermore, this technique allows not only orbital reconstruction through a relatively small incision but also effective reconstruction of large bony defects with ease and shorter operation times. We, therefore, propose this new surgical technique as an effective alternative method for orbital medial wall reconstruction.

## Figures and Tables

**Table 1:** Summary of the enrolled patients' demographics (*p* ≤ 0.05).

	Control group	center	*p* value
Total number of patients	63	48	N/A
Male : Female	42 : 21 (2 : 1)	29 : 19 (1.53 : 1)	0.497
Mean age (years)	41.2 ± 14.8 (range: 18–68)	38.5 ± 16.9 (range: 18–70)	0.378
Mean follow-up (months)	19.3 (range: 12–30)	15.5 (range: 12–22)	N/A

Etiology (% (number))			0.302
Assault	43 (27)	48 (23)	
Traffic accident	19 (12)	21 (10)	
Fall	16 (10)	15 (7)	
Sports injury	13 (8)	10 (5)	
Industrial injury	10 (6)	6 (3)	

**Table 2:** Summary of the preoperative ophthalmic examinations (*p* ≤ 0.05).

	Control group	Combined group	*p* value
Visual acuity	0.87 ± 0.39	0.94 ± 0.45	0.375
Intraocular pressure (mmHg)	14.24 ± 3.62	13.40 ± 3.67	0.230
Extraocular movement dysfunction/Diplopia (%)	25.40 (16/63)	35.42 (17/48)	0.253
Exophthalmometry discrepancy (mm)	1.11 ± 0.69	1.19 ± 0.67	0.505
Fracture area size (cm^2^)	1.75 ± 0.52	1.86 ± 0.66	0.347

**Table 3:** Summary of the results (∗*p* ≤ 0.05).

	Control group	Combined group	*p* value
Exophthalmometry discrepancy (mm)	0.94 ± 0.70	1.05 ± 0.73	0.425
Width discrepancy (mm)	1.55 ± 0.86	1.08 ± 0.69	0.003∗
Orbital volume discrepancy (cm^3^)	2.58 ± 1.40	2.20 ± 1.80	0.209
Length of operation time (minutes)	43.0 ± 7.0	38.3 ± 7.0	0.001∗

## Data Availability

The data used to support the findings of this study are included within the article.
